# Supplementary material for: ECOCAPTURE@HOME: Protocol for the Remote Assessment of Apathy and Its Everyday-Life Consequences
Source: Int J Environ Res Public Health. 2021 Jul 23;18(15):7824. doi: 10.3390/ijerph18157824 (PMC8345445; doi:10.3390/ijerph18157824)

Content of the DAY QUESTIONNAIRE interface

The 24h day report of your chosen day starts on the day before when you go to bed and must be completed at each of the following timepoints: 1/ Going to be, 2/ Getting up, 3/ Breakfast, 4/ Lunch and 5/ Dinner. For each of these five timepoints, we will ask you to focus on your partner ‘s behaviour. For caregivers only, please also complete questions in section “6/ Global day” on your overall perception of the day (for you and your partner) at the end of your chosen day.

1/ Going to bed

- Bedtime :
  Start time: (drop-down menu with hours and minutes)
  End time: (drop-down menu with hours and minutes)

- Your partner went to bed:
  Entirely at your initiative ——•—— Entirely at their own initiative

- Did your partner show signs of tiredness before going to bed?
  Yes, numerous signs of tiredness ——•—— No, no signs of tiredness

2/ Getting up

- Getting up time :
  Start time: (drop-down menu with hours and minutes)
  End time: (drop-down menu with hours and minutes)

- Your partner got up :
  Entirely at your initiative ——•—— Entirely at their own initiative

- Did your partner show signs of tiredness after getting up?
  Yes, numerous signs of tiredness ——•—— No, no signs of tiredness

3/ Petit-déjeuner

- Breakfast time :
  Start time : (drop-down menu with hours and minutes)
  End time : (drop-down menu with hours and minutes)

- How did you perceive your partner’s involvement in activities related to breakfast (preparing drinks/food, clearing up, washing up…) ?
  No involvement at all ——•—— Large amount of involvement

- How did you perceive the usefulness of your partner‘s involvement in activities related to breakfast (preparing drinks/food, clearing up, washing up…) ?
  Completely useless ——•—— Very useful

- If your partner did help you in the activities involved at breakfast, were these:

Completely at your initiative ——•—— Completely at their own initiative

- How would you consider the interactions between you and your partner during breakfast?

None at all ——•—— Very numerous

- Your partner's emotional reactions to your interactions seemed :

Entirely inappropriate ——•—— Entirely appropriate

4/ Lunch

- Lunch time :
  Start time: (drop-down menu with hours and minutes)
  End time: (drop-down menu with hours and minutes)

- How did you perceive your partner's involvement in activities related to lunch (preparing drinks/food, clearing up, washing up…)?

No involvement at all ——•—— Large amount of involvement

- How did you perceive the usefulness of your partner's involvement in activities related to lunch (preparing drinks/food, clearing up, washing up…) ?

Completely useless ——•—— Very useful

- If your partner did help you in the activities involved at lunch, were these:

Completely at your initiative ——•—— Completely at their own initiative

- How would you consider the interactions between you and your partner during lunch?

None at all ——•—— Very numerous

- Your partner's emotional reactions to your interactions seemed:

Entirely inappropriate ——•—— Entirely appropriate

5/ Dinner

- Dinner time :
  Start time: (drop-down menu with hours and minutes)
  End time: (drop-down menu with hours and minutes)

- How did you perceive your partner's involvement in activities related to dinner (preparing drinks/food, clearing up, washing up…)?

No involvement at all ——•—— Large amount of involvement

- How did you perceive the usefulness of your partner‘s involvement in activities related to dinner (preparing drinks/food, clearing up, washing up…) ?

Completely useless ——•—— Very useful

- If your partner did help you in the activities involved at dinner, were these:

Completely at your initiative ——•—— Completely at their own initiative

- How would you consider the interactions between you and your partner during dinner?

None at all ——•—— Very numerous

- Your partner’s emotional reactions to your interactions seemed:

Entirely inappropriate ——•—— Entirely appropriate

6/ Global day (only for caregivers)

1. How did you perceive your partner’s level of apathy (= lack of ability to initiate activities) during the day?

Very low ——•—— Very high

2. How did you perceive the level of burden related to your partner’s care during the day?

Very low ——•—— Very high

3. How did you perceive your overall health state (physical and psychological) during the day?

Very bad health state ——•—— Very good health state

4. Overall, how did you perceive this day?

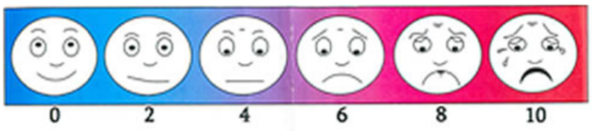

Supplement: Supplementary file 1 [file ijerph-18-07824-s001.zip › File_S1.pdf]
