# Supplementary material for: ECOCAPTURE@HOME: Protocol for the Remote Assessment of Apathy and Its Everyday-Life Consequences
Source: Int J Environ Res Public Health. 2021 Jul 23;18(15):7824. doi: 10.3390/ijerph18157824 (PMC8345445; doi:10.3390/ijerph18157824)
Supplement: Supplementary file 1 [file ijerph-18-07824-s001.zip › File_S2.pdf]

Instructions Manual and Follow-Up Information for the Caregiver in Patient-Caregiver Couples

Couple identifier:
Inclusion date:

During the 4 weeks of follow-up, you will be required to use two pieces of equipment which will enable us to measure, at a distance, certain parameters related to your behaviour: 1/ two ®*Empatica* E4 bracelets (for you and your partner) and 2/ questionnaires accessible via a pre-installed application on your smartphone. The follow-up protocol will start on the first Monday morning following your inclusion into the study, which in your case is on the morning of the ____________ and will end 28 days later, which will be on the morning of the ___________ .

1/ Instructions for using the E4 bracelets

You have in your possession two E4 bracelets: one for you and another for your partner. You should never exchange each other's bracelet. A coloured sticker will enable you to distinguish between the two.

As a reminder:

My bracelet has a _______ sticker

My partner's bracelet has a _______ sticker

You and your partner must wear your bracelet on your non-dominant wrist (left if you are right-handed or right if you are left-handed) as often as possible day and night. Ideally, you should only take off your bracelet:

- when it strongly impacts your activity,
- when you wash yourself, the dishes or when you do hand wash (as bracelets cannot be submerged under water)
- when you charge its battery.

You must turn on the bracelet when you put it on and turn it off when you take it off.

→ How to put on your E4 bracelet, turn it on and off?

1. Place the E4 bracelet on a surface (with the top of the bracelet against the surface)
2. Wrap the E4 bracelet around your non-dominant wrist whilst aligning the side of the bracelet with the electrodes below the gap between your middle and ring finger
3. Close the E4 bracelet whilst keeping it tight enough to ensure the electrodes don't move from their position when performing normal movements (without blocking blood flow or causing any discomfort)
4. To turn on the bracelet, press down on the button for 2 seconds (avoid pressing for longer as this could reinitialise the bracelet). The light on the side of the button will go green when the bracelet is on and then red when the data recording mode is active (then it will go off after 40 seconds). To turn off the bracelet, press on the button again for 2 seconds.

Each of your bracelets is paired with your respective smartphones to enable automatic transfer of your data onto a storage platform. You must therefore have your smartphone with you (either carry it or put it next to you or at least have it in the room where you are) as much as possible during the follow-up 4 weeks.

Every evening (at approximately the same time), you must remember to charge both bracelets' batteries (it takes approximately 1h30 if the battery is completely without charge). It is recommended that you make use of your evening wash to charge your bracelets. When you are wearing your bracelet and the light becomes yellow, this indicates that the battery is nearly out of charge and you must charge it up very quickly.

→ How to charge your E4 bracelets ?

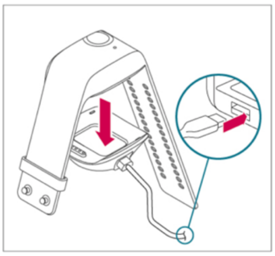

As the image on the right shows, use the USB cable provided with the bracelet and connect one end to the inferior part of the bracelet and the other end (USB end) to a power supply (eg: computer USB port). When the bracelet is charging, the light becomes yellow.

→ How to report a particular event ?

Try to remember to report any events which are potential generators of strong emotions in your partner by tapping the button on your own bracelet very briefly (less then 1 second, without pressing it down). The light will start to flash (red) just after you tap it.

2/ Questionnaire Instructions

You will be required to complete questionnaires 5 times throughout this follow-up procedure via an application pre-installed onto your smartphone: the "DAY QUESTIONNAIRE" interface will be used 4 times and the "MONTH QUESTIONNAIRE" interface will be used once at the end of the 4 weeks. The application will send you notifications to remind you to complete the questionnaires.

The "DAY QUESTIONNAIRE" must be completed once a week during the 4 weeks. Ideally, choose a day in the week when you and your partner are together most of the day (particularly when getting up and going to bed and during meals), without modifying your usual habits. You must therefore complete the "DAY QUESTIONNAIRE":

- on one day between the ______ and the ______
- on one day between the ______ and the ______
- on one day between the ______ and the ______
- on one day between the ______ and the ______

The day report on 24 hours starts at night and must be done throughout the day after at the following moments: 1/ Going to bed, 2/ Getting up, 3/ Breakfast, 4/ Lunch et 5/ Dinner. For these 5 moments, you will have to answer questions about your partner's behaviour. The general day report will occur at the end of the day by completing the "6/ Global day" and answering questions about your general perception of the day (for you and your partner).

The "MONTH QUESTIONNAIRE" must be completed on Day 0 + 28 days, which will be on the morning of the __________ . You must complete 3 questionnaires about your general perception of the past month (about your partner's apathy, impacts for your health and general quality of life).

3/ Support programme

During the 4 weeks of follow-up, you will have 2 telephone appointments with a scientific investigator. These appointments will enable you to express any queries, particularly technical difficulties and ensure that the protocol is running smoothly. Your first telephone appointment will be on the __________ .

In case of any major technical problems or any other issues which may prevent the smooth running of the protocol, you can contact the following scientific investigator on weekdays:
Bénédicte Batrancourt, PhD
Tel : 06 60 44 79 79
E-mail : benedicte.batrancourt@upmc.fr
